# Kin-Mediated Male Choice and Alternative Reproductive Tactics in Spider Mites

**DOI:** 10.3390/biology9110360

**Published:** 2020-10-26

**Authors:** Peter Schausberger, Yukie Sato

**Affiliations:** 1 Sugadaira Research Station, Mountain Science Centre, University of Tsukuba, Ueda, Nagano 305-8577, Japan; sato.yukie.gn@u.tsukuba.ac.jp; 2 Department of Behavioral and Cognitive Biology, University of Vienna, Althanstrasse 14, 1090 Vienna, Austria

**Keywords:** male choice, kin selection, mites, male competition, alternative reproductive tactics, local mate competition, Coolidge effect

## Abstract

**Simple Summary:**

Evolutionary-grounded theories predict that any mating-related behavior should be influenced by the degree of genetic relatedness between the involved interactants. Close genetic relatedness may have beneficial and detrimental effects on evolutionary fitness. To optimize these trade-offs, mates should be able to discriminate kin and non-kin and adjust their behaviors accordingly. Here, we assessed kin-dependent mate choice and adjustment of alternative reproductive tactics (ARTs) in male spider mites. Our experiments suggest that male spider mites can assess kinship of rivals and prospective mates. Mate choice and expression of ARTs (fighting versus sneaking) were driven by direct and indirect fitness benefits arising from the degree of kinship of available mates and rival males competing for access to females. Depending on the social context (choice vs. no-choice, w/wo competition) and achievable fitness benefits, males seemed to use novelty as absolute decision rule and/or comparative evaluation in mate choice. Close kinship among rivals mitigated the males’ fighting propensity and favored adoption of the sneaking tactic. Overall, our study highlights kin-mediated plasticity in male choice and expression of ARTs and emphasizes the importance to consider different contexts and inclusive fitness benefit/cost trade-offs when interpreting mating preferences.

**Abstract:**

Optimal outbreeding and kin selection theories state that the degree of kinship is a fundamental determinant in any mating system. However, the role of kinship in male choice and alternative reproductive tactics (ARTs) is poorly known. We assessed the influence of kinship on male choice and expression of ARTs in two populations of two-spotted spider mites *Tetranychus urticae*. Male spider mites guard premature females, which is an indicator of mate choice, and may conditionally adopt fighting or sneaking tactics to secure access to females. Males competing with kin or non-kin were offered one kin or non-kin female (experiment 1) and single males were presented a choice of kin and non-kin females (experiment 2). Under kin competition, males of both populations were more prone to guard non-kin than kin females at a 3:1 fighter:sneaker ratio. Under non-kin competition, all males were fighters. Under no-choice, males used novelty as indicator of genetic dissimilarity, serving as absolute decision rule for outbreeding. Under choice, comparative evaluation allowed males to preferentially guard females with higher reproductive potential. Overall, our study suggests that male spider mites can assess kinship of rivals and prospective mates. Kin discrimination allows adaptive, context-specific non-random mating preference and adjustment of ARTs.

## 1. Introduction

The classically dominating perspective in sexual selection is female choice and male competition, mainly based on sex-specific differences in parental investment [1]. However, ever-increasing theoretical and experimental work revealed the evolution of male mate choice in many species and circumstances, beyond those predicted by parental investment alone (for review [2]). Male mate choice is expected to evolve if several female mates are simultaneously available, mate availability exceeds male mating capacity, male investment in mating effort is increased (for example, if intrasexual competition is strong and males have to fight to gain access to mates or must express costly courtship or guarding behavior), female mates vary in quality and/or the benefits of being choosy exceed the costs of assessing potential mates [2,3]. Such circumstances may explain the evolution of male mate choice even in highly polygynous species such as in fruit flies *Drosophila melanogaster* [4] and two-spotted spider mites *Tetranychus urticae* [5,6]. Like with female choice, male choice may yield direct and/or indirect (genetic) benefits [2].

Here, we examined the influence of kinship on male choice and male alternative reproductive tactics (ARTs) in two interacting allopatric populations of two-spotted spider mites *Tetranychus urticae*. *Tetranychus urticae* is a globally distributed, highly polyphagous, haplodiploid herbivore with more than 1000 described host plant species [7]. ARTs are distinct ways of achieving reproductive success within the same sex, may occur in both females and males (albeit primarily described for males), and may be genetically determined, conditional or a combination of both [8,9]. Conditional ARTs, which may be irreversible once determined or reversible during life [10], may be influenced by multiple interrelated factors, such as individual state (e.g., age or physiological condition) and environmental variables (e.g., frequency, density and social composition of cohabiting conspecifics or availability of shared resources). Concerning social composition, well-known factors governing ART expression are mate availability, including operational sex-ratio (OSR), and the occurrence of mate competitors and their tactics. Furthermore, kinship has obvious theoretical implications for determination and expression of ARTs [9]. However, while kinship influence on female ARTs has been considered, for example in brood parasitism by birds and insects (for review [11]), kinship influence on male ARTs has been rarely investigated. Observational examples come from ants [12] and fig wasps [13] having dichotomous male ARTs, wingless fighters and winged dispersers. Foitzik et al. (2002) [12] observed only winged males in single-queen headed, and thus more closely related, ant colonies but both male ARTs, winged and wingless, in polydomous (multiple queens) colonies. Cook et al. (2015) [13] concluded on kin-selected maternal control of competition avoidance among fighter sons in fig wasps, because of mothers laying only one fighter son per fig. Whether and how kinship among competing males, and between males and their prospective mates, affects the expression of ARTs is unexplored for any animal.

We conducted two experiments to scrutinize mate choice and ARTs of male *T. urticae* in presence or absence of competing males from their own or another population and presented mates from their own or another population. Due to long-term closed rearing in the laboratory and allopatric origin of the two populations, members of the same population were considered kin, while members of different populations were considered non-kin (see Section 2.1 for details). In detail, we assessed the influence of kinship among male competitors and between males and premature females on male guarding behavior and the expression of ARTs. *Tetranychus urticae* males guard females in their final premature phase, which is a quiescent stage called teleiochrysalis (subsequently called T-females). Guarding behavior is highly important for paternity success, because of first male sperm precedence [14,15,16,17], and is thus a suitable indicator of male choice. Regarding ARTs, *T. urticae* males may adopt fighting or sneaking tactics before and during pre-copulatory guarding behavior [18,19,20]. ARTs of *T. urticae* males are conditional and reversible [18,19]. Fighters fight with other males to gain access to T-females and to defend their guarding position; sneakers do not fight but build on speed (i.e., being quick to start guarding) and crypsis towards other males (preventing them from being fought) to succeed in gaining access to T-females and keeping their guarding position [18,19,20].

We hypothesized that male choice should be non-random (positive or negative assortative) driven by direct and/or indirect benefits. Assortative mating is non-random mating based on phenotypic similarity between mates; positive assortative describes preferential mating between phenotypically similar individuals; negative assortative (also called disassortative) describes preferential mating between phenotypically dissimilar individuals [21,22]. Direct (material) benefits may arise to males from higher reproductive potential of the chosen mate, which may be linked to genetic relatedness or not. Indirect benefits may arise from inbreeding by increased level of genetic relatedness or, conversely, from outbreeding by enhanced genetic quality of offspring. Mate choice for indirect benefits presupposes the presence of phenotypic traits reflecting genetic relatedness/similarity and kin discrimination ability. *Tetranychus urticae* has been suggested to possess kin discrimination abilities [6,23]. Kin selection [24,25] should promote inbreeding tolerance or preference [26,27] and positive assortment. In contrast, risk of inbreeding depression (for review [28]; for *T. urticae* [6,29]) should promote inbreeding avoidance and negative assortment. The assumption of assortative mating is based on the premise that the cost/benefit trade-offs of in- and out-breeding do not completely counterbalance each other. Additionally, we hypothesized that *T. urticae* males should adjust their ARTs to the presence of kin versus non-kin competitors. Kin selection, in particular also inferences arising from local mate competition (LMC) theory [30], predicts that male-male competition should be adjusted to the level of rival relatedness and be fiercer among non-kin than kin. Regarding conditionally expressed high and low energy-investing ARTs, non-kin male competition should thus favor the adoption of the more aggressive high energy-investing tactic, which is in *T. urticae* the fighter phenotype. Since male fights are costly and may even cause lethal physical injuries ([16]; personal observations), the fighting tactic should occur less likely among kin than non-kin competitors.

## 2. Materials and Methods

### 2.1. Experimental Animals

*Tetranychus urticae* (red form) used in experiments came from two allopatric populations, called Y and G, reared in the laboratory. The Y-population had been founded about 5 years before conducting the experiments by specimens obtained from Koppert B.V, NL, while the G-population had been founded about 2 years before the experiments by specimens collected on tomato plants in Iida, Nagano, Japan [18,31]. Either population was founded by 50 to 100 females and maintained at fluctuating sizes of hundreds to thousands of individuals. In the laboratory, both populations were separately kept on detached primary leaves of common bean, *Phaseolus vulgaris*, resting on water-saturated cotton pads inside Styrofoam trays. Assuming a generation time of about 12 to 15 days [32], the Y- and G-populations had been subjected to closed rearing for >120 and >50 generations, respectively, before conducting the experiments. Multi-generational closed rearing and haplodiploidy (arrhenotoky) allowed considering individuals from the same population as kin and from different populations as non-kin. The estimated coefficients of inbreeding (*F_t_*; t is the number of generations) were *F*_50_ = 0.22 to 0.40 for the G-population and *F*_120_ = 0.45 to 0.70 for the Y-population at 100 to 50 foundresses, respectively [33]. Rearing technique, abiotic conditions and host plant quality were exactly the same for both populations.

To obtain virgin males for experiments, T-females were randomly withdrawn from the Y- and G-populations and placed in groups of 10 (all either Y or G) on single detached primary bean leaves resting on water-saturated cotton pads inside Styrofoam trays. After molting to adult, unmated females were allowed to oviposit for 5 d (due to arrhenotoky unmated females exclusively produce haploid sons) and their sons allowed to develop to the teleiochrysalis (T) stage. T-males were collected and placed in groups of five on circular leaf discs (1.5 cm), resting on moist cotton pads inside acrylic cages, to molt to adult within 24 h. Adult males <24 h old were used in the experiments.

Bean plants were grown and experimental units stored inside climate chambers at 25 ± 1 °C and 16:8 h light:dark photoperiod.

### 2.2. Male-Male Competition, No-Choice

Groups of six virgin males from either the Y- or G-population or from both populations combined (YG), were presented a T-female coming from either their own (kin) or the other population (non-kin), resulting in six treatments: six Y-males presented a Y-female (Y/Y), six Y-males presented a G-female (Y/G), six G-males presented a Y-female (G/Y), six G-males presented a G-female (G/G), three Y- plus three G-males presented a Y-female (YG/Y), and three Y- plus three G-males presented a G-female (YG/G). Y- and G-males of the latter two treatments (YG/Y and YG/G) were marked with different tiny water color dots on their dorsal sides (randomly assigned) before use in the experiment to make them distinguishable. To start the experiment, single T-females of similar age and size from the Y- and G-populations were randomly withdrawn from the rearing units (avoiding T-females in the silvery phase because being close to emergence) and singly placed on fresh leaf discs inside acrylic cages. Each disc then received six males from either the Y- or G-population or three males from the Y-population plus three males from the G-population. Care was taken to randomly combine males that were unfamiliar with each other, i.e., which had matured on different discs. Male guarding behavior and ambulating activity was monitored every 45 min until occurrence of two guarding males or for a maximum of 4.5 h. The reproductive tactic of the guarding males was determined by the “brush test” [18]; the first guarding male was removed from the disc after determining his ART. The “brush test” consists of randomly picking up a male from behind from the rearing, using a moistened brush (marten’s hair, size 0), and allowing the lifted male to contact the guarding male with his first pair of legs. Guarding males assuming the threatening posture, i.e., raising and spreading their first pair of legs, are considered fighters, whereas guarding males not responding to the contacting male are considered sneakers [18]. Each treatment was replicated 24 to 35 times.

### 2.3. Male Choice, No Competition

Single virgin males from the Y- or G-population were given a choice between two T-females, one from the Y-population and the other from the G-population, resulting in two treatments. To start the choice experiment, two T-females of similar age and size (avoiding T-females in the silvery phase because being close to emergence), one from the Y-population and the other from the G-population, were placed at an inter-individual distance of 0.5 cm on fresh leaf discs and either one virgin Y-male or one virgin G-male added. The discs were monitored every 45 min until first guarding of either the Y- or G-female by the male. Each treatment was replicated 71 (Y) and 96 (G) times.

### 2.4. Body Size Measurements

Body size of males from the Y- and G-population used in experiments was estimated by embedding adult males in a droplet of Hoyer’s medium on a microscope slide and covering the droplet by a cover slip. Subsequently, the slides were placed on a heating plate at 40 °C for 2 weeks. Using a digital USB camera (AnMo Electronics Corporation, New Taipei City, Taiwan) attached to a phase contrast microscope equipped with an object micrometer (Leica DM6000B, Wetzlar, Germany), body length was estimated by taking the mean of the right- and left-sided distances between the bases of the prodorsal setae P1 and dorsal setae D4 (F1); body width was estimated by taking the distance between the left- and right-sided bases of prodorsal setae P3 (nomenclature of dorsal setae [34]). A total of 26 Y-males and 25 G-males were measured.

Body size of T-females from the Y- and G-population (N = 14 for each population) used in experiments was measured on-screen using a monitor attached to a digital microscope (Leica DMS1000, Wetzlar, Germany). Live T-females (avoiding the silvery phase) were randomly selected from the rearing and placed ventral side down on a flat bean leaf surface. Body length and width were estimated on-screen from the ellipsoid contour of the female, using the two-point vector tool of Leica DMS1000, by taking the longest distance from the prodorsal end (in between P1 setae) to the opisthosomal end of the idiosoma and the widest distance from the right- and left-sided lateral edges of the dorsum of the idiosoma.

### 2.5. Statistical Analyses

IBM SPSS Statistics 25 (Armonk, NY, USA) was used for all statistical analysis. In the male-male competition no-choice experiment, we use generalized linear models (GLM; binomial distribution, probit link, counts of events) to analyze the influence of female and male population-of-origin on the number of guarding and ambulating males. A GLM (binomial distribution, logistic link) was used to compare the number of Y- and G-males succeeding in obtaining the guarding position in mixed, Y and G, groups. Male ART ratios (fighter to sneaker) were analyzed using two-sided Fisher’s exact tests: first, the influence of T-female origin, Y or G, on the ART ratio (fighter to sneaker) across males was assessed; then, the ART ratios (fighter to sneaker) of males of pure Y-groups, pure G-groups and mixed, Y and G, groups across T-female origins were compared. In the choice experiment, we used separate GLMs (binomial distribution, logistic link) to compare male preference for the kin or non-kin female between male origins (Y and G) and within each male origin. Body length and width of Y- and G-males and Y- and G-T-females were compared by two-sided T-tests for independent samples.

## 3. Results

### 3.1. Male-Male Competition, No-Choice

**Figure 1:**
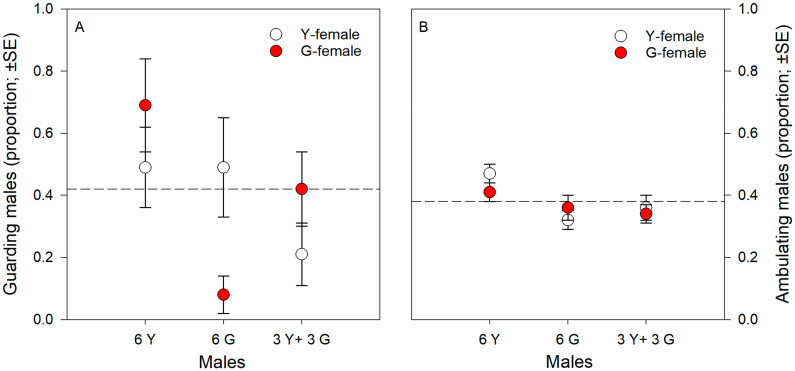
Kin-mediated guarding propensity (**A**) and ambulating activity (**B**). Guarding propensity and ambulating activity of males from the Y- and G-populations presented one kin (own population) or non-kin (other population) female. Males were held in groups of six (either 6 Y or 6 G or 3 Y + 3 G) together with a teleiochrysalis female (either Y or G) on a leaf disc and their guarding behavior and ambulating activity observed until two guarding events occurred for 4.5 h at maximum (N is 24 to 35 discs per treatment). First guards were removed after detection. Broken lines represent the grand average.

**Figure 2:**
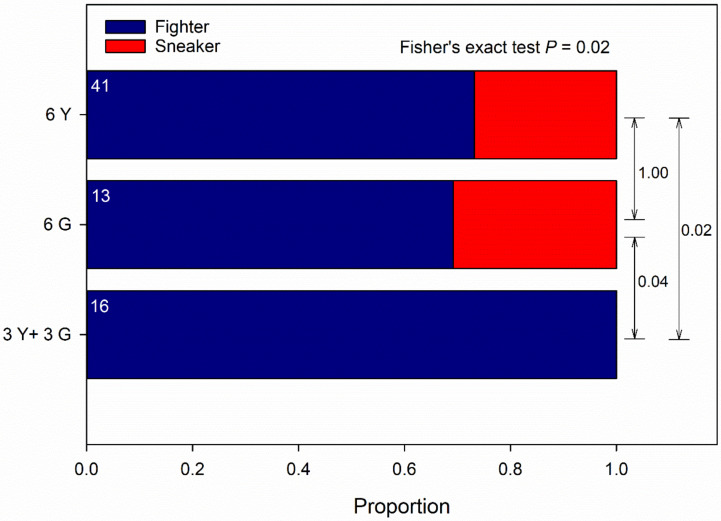
Kin competition-mediated alternative reproductive tactics (ARTs). ARTs by males from the Y- and G-populations presented one kin (own population) or non-kin (other population) female (data of presenting a kin or non-kin female were pooled because kinship to the female did not influence male ARTs; Fisher’s exact test *p* = 0.20). Males were held in groups of six (either 6 Y or 6 G or 3 Y + 3 G) together with a teleiochrysalis female (either Y or G) on a leaf disc and monitored for the occurrence of guarding behavior and the ART of the first two guards for 4.5 h at maximum. First guards were removed after determining their ART. *P*-values refer to Fisher’s exact test; *p*-values of multiple pairwise comparisons were adjusted by the Benjamini–Hochberg method (FDR); numbers inside bars refer to the number of observed guarding males.

Guarding propensity was significantly influenced by male origin (Wald χ_2_^2^ = 8.953, *p* = 0.01) and the interaction between male and T-female origin (Wald χ_2_^2^ = 8.500, *p* = 0.01) but not by T-female origin as main effect (Wald χ_1_^2^ = 0.170, *p* = 0.68) (GLM; binomial distribution, probit link, counts of events). Males of pure Y groups had a greater guarding propensity than males of pure G groups and males of mixed, Y and G, groups; the significant interaction term indicates that males of pure Y and G groups had a greater guarding propensity towards non-kin than kin T-females; the guarding propensity of males of mixed, Y and G, groups was biased towards G-T-females (Figure 1A), which was due to 14 out of 16 males succeeding in securing the guarding position being Y males (GLM; binomial distribution, logistic link; Wald χ_1_^2^ = 6.626, *p* = 0.01). Male ambulating activity was significantly influenced by male origin (Wald χ_2_^2^ = 9.849, *p* = 0.007) but not by T-female origin (Wald χ_1_^2^ = 0.079, *p* = 0.77) and the interaction between male and T-female origin (Wald χ_2_^2^ = 1.943, *p* = 0.37) (GLM; binomial distribution, probit link, counts of events). Males of pure Y groups were more active than males of pure G groups and males of mixed, Y and G, groups (Figure 1B). Male ART ratio (fighter to sneaker) was unaffected by T-female origin across male groups (Fisher’s exact test; two-sided *p* = 0.20) but differed significantly among male origins across T-female origin (two-sided *p* = 0.02). Around 25 to 30% guarding males of pure Y or G groups adopted the sneaking tactic, whereas all guarding males of mixed, Y and G, groups were fighters (Figure 2).

### 3.2. Male Choice, No Competition

**Figure 3:**
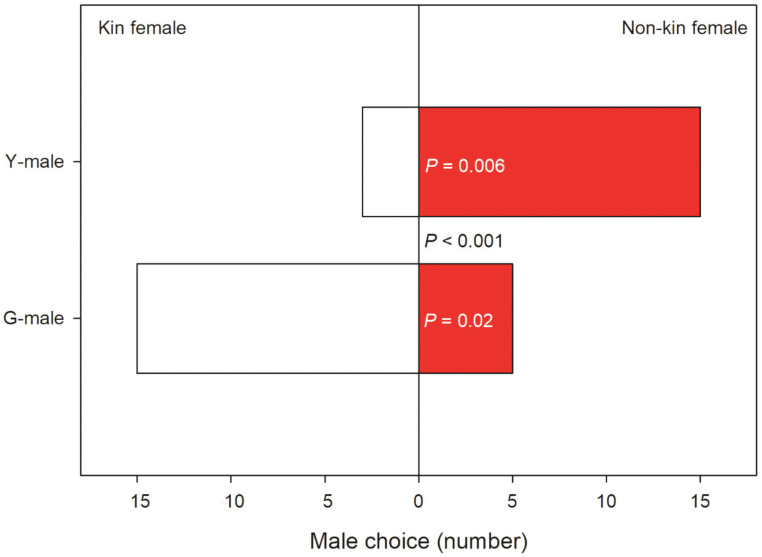
Kin-mediated mate choice. Mate choice by Y- and G-males presented two teleiochrysalis females, one from their own population (kin) and one from the other population (non-kin) (N is 18 for Y and 20 for G). The *p*-value between bars refers to the comparison of choice by Y- and G-males; *p*-values inside bars refer to choice between kin and non-kin females within Y- and G-males.

**Figure 4:**
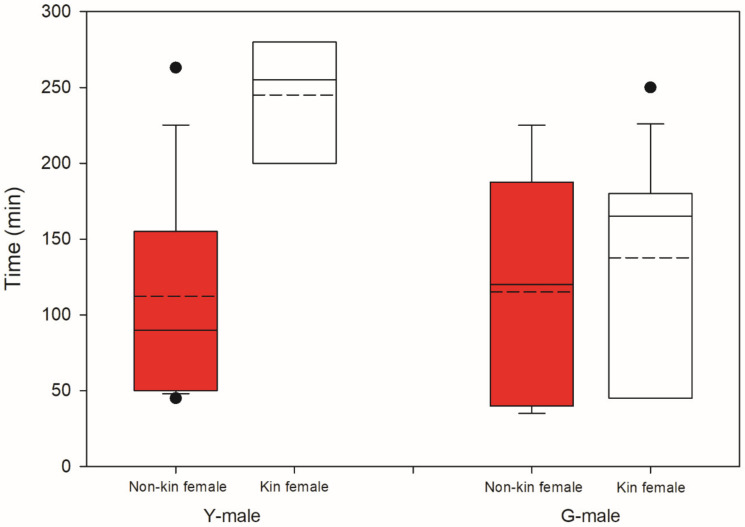
Kin-mediated guarding latency. Time elapsed until mate choice (guarding latency) by Y- and G-males presented two teleiochrysalis females, one from their own population (kin) and one from the other population (non-kin) (N is 18 for Y and 20 for G). Solid lines inside boxes indicate the median, broken lines indicate the mean; boxes show the 50% interquartile range; whiskers show the lower and upper quartiles; dots represent the outliers.

Male mate preference differed significantly between male origins (GLM; binomial distribution, logistic link; Wald χ^2^ = 12.455, *p* < 0.001). GLM (binomial distribution, logistic link) within each male origin revealed that Y-males given a choice between a kin T-female (from their own population) and a non-kin T-female (from the other population) preferentially guarded the non-kin T-female (Wald χ^2^ = 7.572, *p* = 0.006). In contrast, G-males given a choice between a kin and a non-kin T-female preferentially guarded the kin T-female (Wald χ^2^ = 4.900, *p* = 0.02) (Figure 3). All guarding males but one G-male preferentially guarding a kin T-female were fighters. Time elapsed until mate choice (guarding latency) was influenced by male origin (Wald χ^2^ = 4.065, *p* = 0.04), T-female origin (Wald χ^2^ = 8.991, *p* = 0.003) and the interaction of these two factors (Wald χ^2^ = 4.512, *p* = 0.03). The significant interaction term indicates that Y-males guarded kin T-females later than they guarded non-kin T-females, whereas the guarding time by G-males did not differ between kin and non-kin T-females (Figure 4).

### 3.3. Body Size

Body size (µm; mean ± SE) of adult males did not differ between Y-males (length = 183.85 ± 1.31, width = 152.11 ± 1.29) and G-males (length = 186.05 ± 1.80, width = 155.43 ± 1.68) (*t*-test; length: T_49_ = 0.993, *p* = 0.326; width: T_49_ = 1.569, *p* = 0.123). Body size (µm; mean ± SE) of T-females did not differ between Y-females (length = 294.89 ± 3.59, width = 181.20 ± 1.03) and G-females (length = 293.55 ± 4.61, width = 180.20 ± 1.82) (*t*-test; length: T_26_ = −0.230, *p* = 0.820; width: T_26_ = −0.478, *p* = 0.636).

## 4. Discussion

Our study demonstrates that two-spotted spider mite males choose their mating partners non-randomly and distinguish between (kin) females from their own population and (non-kin) females from an allopatric population. In the presence of kin competitors and without choice, males of both populations were more eager to guard non-kin females than kin females. Under kin competition, about 25 to 30% adopted the sneaking tactic and 70 to 75% were fighters. Under non-kin competition, all males adopted the fighting tactic; Y-males were superior competitors to G-males independent of kinship to the available female. In absence of competitors and with choice, males of both populations had a strong preference for G-females, i.e., G-males assorted positively and Y-males assorted negatively. All males but one adopted the fighting tactic in the choice experiment.

### 4.1. Proximate Mechanisms

Guarding spider mite males are mainly attracted (arrested) by pheromones released by T-females [35,36]. Body size can play a role in male-male competition, with larger males usually winning fights over smaller ones [15], and male choice because of a positive correlation between female body size and fecundity [32]. In our experiments, body size differed neither between males nor between T-females from the Y- and G-populations. Therefore, males based their guarding decisions most likely on perception of pheromonal differences between kin and non-kin females. Similarly, differences in ART ratios of males competing with kin and those competing with non-kin were likely mediated by perception of differences in chemical profiles. Ambulating activity of Y- and G-males did not vary with the origin of the presented female, which indicates that differences in guarding of Y- and G-females were due to changes in male arrestment rather than in mate search. Overall, discrimination by Y- and G-individuals must have been based on population-specific pheromone labels and represented either innate discrimination ability or phenotype matching, i.e., learned recognition based on previous encounters with male population members, and subsequent formation of a generalized population-specific template, or by self-referencing [37]. All males used in experiments were virgin and did not encounter any female (neither juvenile nor adult) before the experiment. Males grouped on leaf discs in the no-choice experiment were unfamiliar to each other. Since all environmental variables during rearing and experimentation, such as host plant species and quality, were exactly the same for both populations, we assume inherent population-specific labels [31].

### 4.2. Male-Male Competition, No-Choice

Assuming that guarding propensity and ART ratio reflect the intensity of competition [20], males competed more strongly for non-kin females and when competing with non-kin males. In general, intense competition should increase male mating efforts [2]. Under kin competition, both Y- and G-males increased their mating efforts in presence of a non-kin female, which was evident in greater guarding propensity towards non-kin than kin females. Non-kin females were, probably due to novelty, perceived as more attractive/stronger stimuli than kin females. Novelty may be used as indicator of genetic dissimilarity. Going for novel phenotypes may thus constitute an outbreeding mechanism to avoid inbreeding depression and possibly provide indirect benefits via enhanced offspring quality. Enhanced response by virgin males to novel mates (novelty regarding the population-specific label) is similar to the Coolidge effect [38,39] or may be called a Coolidge effect *sensu lato. Sensu stricto*, the Coolidge effect refers to an enhanced response to novel mates as compared to the response to (directly) familiar mates [38]. Greater propensity to choose novel phenotypes is adaptive if it increases the likelihood to inseminate multiple mating partners, for example in animals with poor abilities to discriminate among phenotypically similar individuals (such as those coming from the same population), or between fertilized and unfertilized females of the same population (for *T. urticae* [40]).

Non-kin competition increasing male mating efforts relative to kin competition was evident from the exclusive occurrence of fighters in mixed, Y and G, groups. Adopting the fighter mode is competitively advantageous to adopting the sneaker mode because providing greater direct fitness benefits to both the male and his mate. Females mated to fighters produce more daughters than females mated to sneakers [20], which enhances fitness of both female and male spider mites [41]. Co-occurrence of both reproductive phenotypes under kin competition versus sole occurrence of the more aggressive fighter phenotype under non-kin competition appears kin-selected [30], relaxing local competition among kin, by some being in the sneaking mode, yet intensifying competition among non-kin, by all being in the fighting mode. The observed alteration of the ART ratio in mixed, Y and G, groups provides evidence that, at the same male density and operational sex ratio [19], social composition (here kinship) drives the expression of ARTs by male spider mites.

### 4.3. Male Choice, No Competition

In the choice experiment, mate preference by both Y- and G-males for G-females was aligned with achievable direct benefits. Preferential guarding of G-females by both Y- and G-males is adaptive because of higher fecundity of G- than Y-females; G-females mated to Y- or G-males produce 40 to 70% more eggs than Y-females mated to Y- or G-males [31]. In general, direct benefits are considered stronger drivers of mate choice than are indirect benefits [2,4,42,43]. Nonetheless, Y-males mating with non-kin G-females may additionally gain indirect benefits if outbreeding enhances the genetic quality of offspring [3]. In contrast, G-males could indirectly benefit from kin mating because of increased genetic relatedness of offspring and favoring kin as mates [26,27]. Large difference in perceived quality of the two available T-females may explain why all males but one showed high mating efforts and adopted the fighting tactic. Variation in female quality is a well-known driver of male mating effort [2,43]. Fighting reduces the capacity to mate more strongly than does sneaking (fighting represents a higher investment in mating effort than does sneaking). Therefore, fighters should be choosier than sneakers. The fact that, in the choice experiment, all but one guarding males were fighters supports this assumption. G-males going for (the preferred) kin females started guarding at about the same time as those going for non-kin females, which points at comparative evaluation [44] and the need to weigh conflicting signals of obtaining indirect benefits by outbreeding and higher direct benefits by kin mating. In contrast, Y-males being much quicker to start guarding (the preferred) non-kin than kin females points at the use of an absolute decision rule [45], possibly complemented by comparative evaluation, and aligned signals of achievable direct and indirect benefits.

## 5. Conclusions

Our study suggests that mate choice and ARTs by male spider mites depend on the social context [44,45,46,47] and are driven by direct and indirect benefits. In the no-choice experiment, evaluation of the female by the males likely occurred via comparing the female’s signal to an internal population-specific template, an absolute decision rule [45]. In contrast, the choice experiment allowed direct comparison of the two females’ signals; males went for the more attractive/stronger signal, likely based on comparative evaluation (applicable to both Y- and G-males [44]) and/or novelty (applicable to Y-males). Y-males showed aligned but G-males contrasting mate preferences in the no-choice male competition and choice without male competition experiments. Plasticity in male mating preference in dependence of the social context, such as presence of rivals, has also been observed in fruit flies *Drosophila melanogaster* [48] and mosquito fish *Gambusia holbrooki* [49]. The consistent preference of Y-males for G-females may have been due to a higher risk of inbreeding depression in the Y-population, which had been subjected to closed rearing for longer time (>120 generations) than the G-population (>50 generations). Novelty may have been used as proxy of genetic distance and may explain the increased guarding propensity (mating effort) towards non-kin females in the no-choice experiment. In the choice experiment, mate preference was primarily driven by direct benefits, outweighing novelty effects in G-males. Overall, our study highlights plasticity in male choice and expression of ARTs and emphasizes the importance to consider different experimental designs and inclusive fitness benefit/cost trade-offs when interpreting mate preferences and adjustment of ARTs.
